# Training Curriculum, Skills, and Competencies for Global Health Leaders: Good Practices and Lessons Learned

**DOI:** 10.5334/aogh.3212

**Published:** 2021-07-12

**Authors:** Meike J. Schleiff, Patrick Mwirigi Mburugu, John Cape, Rama Mwenesi, Nathanael Sirili, Sean Tackett, David P. Urassa, Bhakti Hansoti, Yohana Mashalla

**Affiliations:** 1 Johns Hopkins School of Public Health; 2 STAR; 3 Jomo Kenyatta University of Agriculture and Technology, School Of Medicine; 4 Afya Bora Consortium; 5 Global Health Corps; 6 University of Michigan School of Nursing; 7 School of Public Health and Social Sciences, Muhimbili University of Health and Allied Sciences, Tanzania; 8 Johns Hopkins Bayview Medical Center; 9 School of Public Health, Muhimbili University of Health and Allied Sciences, Tanzania; 10 Johns Hopkins School of Medicine; 11 School of Health Sciences, University of Botswana, Gaborone, Botswana

## Abstract

**Objectives:**

This paper aims to depict unique perspectives and to compare and contrast three leadership programs for global health in order to enable other training institutions to design impactful curricula.

**Methods:**

We purposively selected three global health training programs. We used a six-step curriculum development framework to systematically compare the curriculum process across programs and to identify best practices and factors contributing to the impact of each of these programs.

**Findings:**

All three fellowship programs undertook an intentional and in-depth approach to curriculum development. Each identified competencies related to leadership and technical skills. Each defined goals, though the goals differed to align with the desired impact of the program, ranging from improving the impact of HIV programming, supporting stronger global health program implementation, and supporting the next generation of global health leaders. All programs implemented the curriculum through an onboarding phase, a delivery of core content in different formats, and a wrap-up or endline phase. During implementation, each program also utilized networking and mentoring to enhance connections and to support application of learning in work roles. Programs faced overlapping challenges and opportunities including funding, strengthening partnerships, and finding ways to engage and support alumni.

**Conclusions:**

Local ownership of programs is critical, including tailoring curricula to the needs of specific contexts. Strong partnerships and resources are needed to ensure program sustainability and impact.

**Key Takeaways:**

- Global health competencies and curricula should be linked to local health system needs and contexts where learners are working.
- Emphasizing both individualistic and collectivist approaches to learning is important in engaging and supporting diverse global health learners.
- Emphasizing mentorship and opportunities to apply learning in contexts where learners are working is important in order to provide support to learners as they work to integrate what they are learning into their professional roles and activities.
- Partnerships and resources—including donor support—are essential to implement and sustain robust leadership curricula and to provide opportunities for experiential and didactic learning.

## Background

Global health has been defined as “the area of study, research and practice that places a priority on improving health and achieving equity in health for all people worldwide” [1,2]. The goal of global health is worldwide health improvement, reduction of disparities, and protection against global threats [3]. In recent years, the field of global health has experienced exponential growth with significant investments and new partnerships between entities in high-income countries (HIC) and low- and middle-income countries (LMIC) [4]. The changing landscape of global health has also spurred the need to champion an increased emphasis on interprofessional approaches to health service delivery and the cultivation of leadership skills to build local leadership capacity [5].

A number of groups have been developing guidance and programs to effectively build needed leadership capacity at country and local levels. There has also been a broad shift towards competency-based education, which means focusing on applying and assessing skills and knowledge rather tracking learning by time spent in a classroom [6]. Multiple competency frameworks for global health and public health have been developed [7,8,9]. For example, the Consortium for Global Health (CUGH) developed interprofessional competencies for global health that can be adopted as guidelines when developing training curricula with different scopes and available resources [8]. The process of developing global health competencies and curricula is often insufficiently inclusive of input from host country health professionals and furthermore fails to take adequate account of local health contexts. In addition, the methods applied and resources available for meaningfully assessing global health curricula are frequently inadequate [10,11].

In its basic format, “curriculum” refers to the lessons and academic content taught in a school or in a specific course or program [12,13]. There are several ways that curricula can be focused and organized, including 1) subject-centered, 2) learner-centered, and 3) problem-centered design. For the purposes of this paper, we take a learner-centered approach. Learner-centered teaching posits that faculty members should focus their efforts on what students need to learn, tailoring learning to the priorities of specific target audiences [14].

In order to bridge current global health leadership training gaps, several global health training programs have developed competency-based curricula targeting different groups of health professionals. One such program is the Afya Bora Consortium Fellowship, established in response to a “Call For Action” in improving leadership in global health programs. This program is targeted towards senior health professionals (having more than five years professional experience) from across the fields of medicine, nursing, and public health, to fill gaps in leadership and management of HIV/AIDS programs [15]. Sustaining Technical and Analytic Resources (STAR) is a program established in response to the recognition by USAID that more explicit emphasis is needed on capacity strengthening and leadership development for leading technical professionals, as well as the teams and organizations they work, which are the target audiences for STAR. The program aims to develop the next cadre of global health technical professionals by bolstering traditional work-based fellowships with dedicated time for leadership development, focused learning activities, and linkages to academic resources [16]. Global Health Corps (GHC) was established to foster a diverse, highly skilled, and tightly-networked community of leaders who work together across disciplines in order to strengthen health systems and targets young and diverse global health professionals. GHC’s unique co-fellow model matches two fellows—one national and one international fellow—with health organizations for a 13-month fellowship. During this fellowship, they receive substantial leadership and management training, coaching, and technical mentorship.

Development of leadership capacity with comprehensive skills to navigate the challenging health care systems in LMIC and HIC has resulted in several innovations in global health leadership training that have incorporated input from host countries and have been suited for local contexts. However, there is a limited understanding of the unique features, successes, and challenges of such programs.

## Objective

This paper aims to depict unique perspectives from representatives to compare and contrast three leadership programs for global health in order to enable other training institutions to design impactful curricula.

## Methods

We purposively selected a set of three global health leadership training programs (The Afya Bora Consortium Fellowship in Global Health Leadership, The Sustaining Technical and Analytic Resources (STAR) Project, and Global Health Corps (GHC)). We aimed to illustrate a range of educational models for which we have extensive first-hand experience. All of the programs had the following criteria in common:

- The program must not be solely clinically focused and must aim to train professionals to lead and manage public health programming around the world.
- The program must include an explicit emphasis on leadership development.
- The program must include a focus on LMIC-based participants and on strengthening capacity for leadership in LMICs, particularly across the African continent.
- The program must not rely solely or predominantly on bringing participants to the US or Europe for study and work opportunities, but rather aim to reach and support them in gaining skills and expertise within the context they are working in around the world.
- The program has been in operation long enough to have evidence and experience available.

### Framework for comparing programs

**Figure 1:**
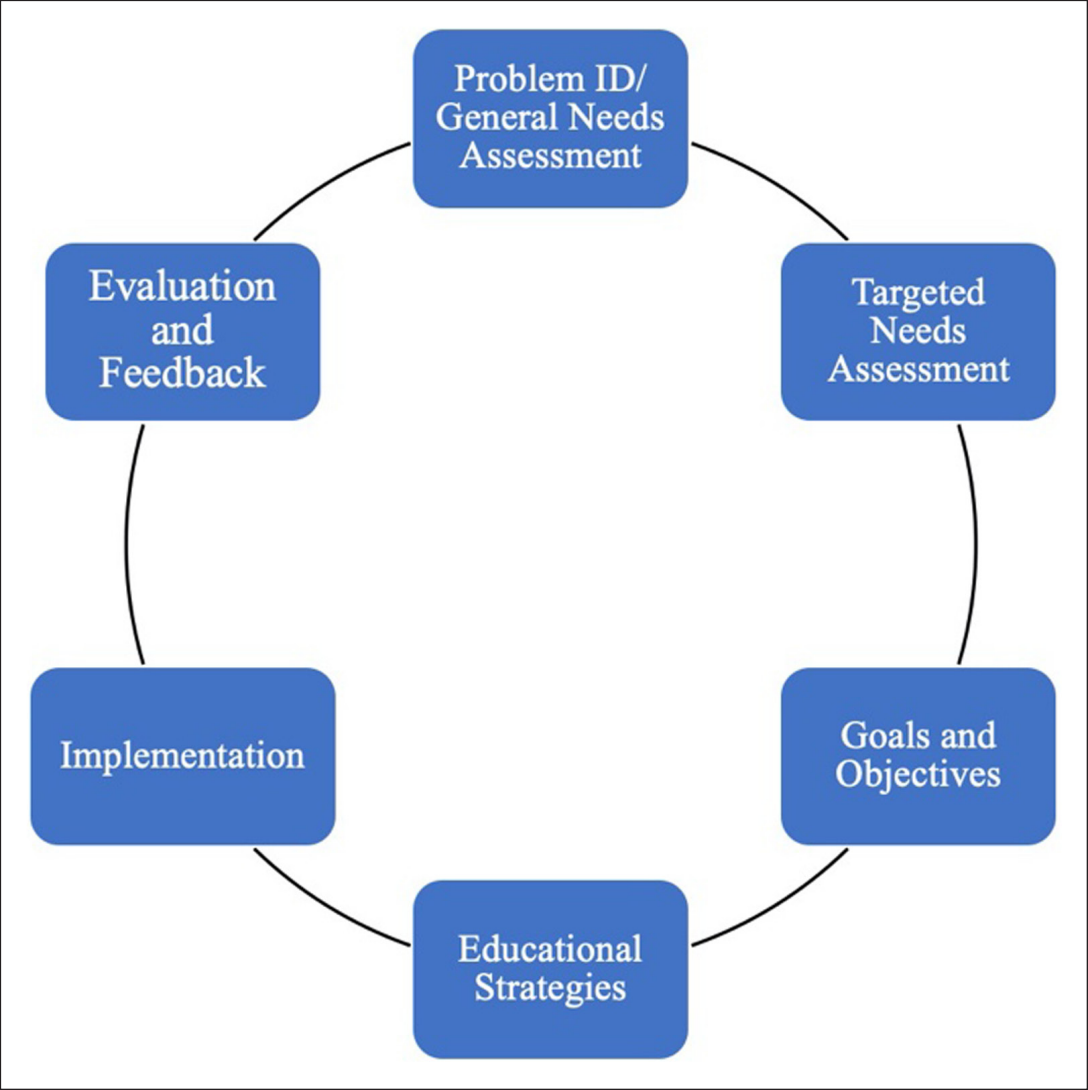
Six-Step Curriculum Development Framework.

We adapted a six-step curriculum development process [13] to inform our framework for comparing the selected training programs. This curriculum development process, described in ***Figure 1***, begins with problem identification and a general needs assessment (top) and follows an iterative cycle of planning, implementation, and evaluation; the process was developed for medical education programs but has been used widely across the Johns Hopkins University, as well as with other training programs for other sectors.

### Data Analysis and Synthesis

Data on the programs’ curricula development procedures, target population, structure, implementation—including delivery mode, assessment, monitoring and evaluation procedures, expected outcome, and impact—were collected and reviewed. Trustworthiness and consistency of data presentation was achieved through regular reviews and discussion among team members and members of the program working groups. A comparative analysis of the training programs was made to elucidate key outcomes of each program.

## Findings

### Descriptions and comparison of global health leadership training programs

The following program descriptions provide an overview of each program’s genesis and aims, including how each was adapted to the needs of its particular target audience.

#### 1. The Afya Bora Consortium Fellowship in Global Health Leadership

In 2011, the Afya Bora Consortium, comprising nine academic medical institutions (five in Africa and four in the United States) started implementing an interprofessional global health leadership training program for participants from across Africa and the US. The overarching goal was to fill in the gaps in leadership for the HIV/AIDS programs [15]. The fellowship program is uniquely designed to ensure trainees acquire leadership skills that are not part of traditional medical, nursing, and public health curricula. The program imparts technical expertise in planning, designing, implementing, monitoring, and evaluating health interventions projects and organizational strategies to prepare participants for positions in governmental, non-governmental, clinical, and academic health institutions [17]. To date, Afya Bora has trained 146 health professionals. Among the fellows, 52% are doctors, 44% are nurses, and 4% are public health professionals. The strength of the Afya Bora Consortium fellowship lies in the diversity of its curricula and the fact that it is aligned with leadership gaps in LMIC in Africa, the interprofessional nature of recruited fellows, the north-south and south-south collaboration, module delivery, mentorship, and networking.

#### 2. The Sustaining Technical and Analytic Resources (STAR) Project

STAR was established to build on several decades of experience managing fellowships at USAID, via the well-established Global Health Fellows Program (GHFP). The impetus for establishing STAR was a move to transition from technical assistance only to capacity strengthening and leadership development—especially within LMIC settings. STAR fellows can have from 2 to 15+ years of professional experience; 50% are based abroad, and a significant portion boast advanced degrees. STAR aims to develop the next cadre of global health technical professionals by bolstering traditional work-based fellowships with dedicated time for leadership development, focused learning activities, and linkages to academic resources. As such, the STAR learning developed a high impact individualized learning curriculum that incorporates principles of deliberate practice and competency-based training to support learning and capacity development across a wide breadth and depth of participants. The goals of the STAR learning program are as follows:

- Enable highly skilled public health workers to fulfill technical roles in local global/public health programming
- Train fellows that can perform at practicing level, or higher, across all eight core competencies
- Help fellows develop skills and strategies for knowledge sharing

STAR is designed to respond to the challenges of lack of protected time available for learning, especially for senior professionals, as well as lack of resources dedicated to leadership training in global health.

#### 3. Global Health Corps (GHC)

Since its founding in 2009, GHC has recruited and trained over 1,000 young leaders committed to transforming health systems and placed them with more than 150 global health organizations in the USA and east and southern Africa, including Ministries of Health, NGOs, and grassroots organizations. This paid fellowship program matches the capacity needs of partner organizations with talented individuals seeking to build careers in global health. GHC complements fellows’ work experience with a robust curriculum and coaching focused on leadership and management skills. The curriculum is built around four pillars—systems thinking, design thinking, authentic leadership, and collective leadership—and is designed to complement the technical learning fellows acquire through their workplace. GHC’s co-fellow model and cohort-based learning ensures that fellows can engage with unique and diverse perspectives and also develop a rich network of peers across disciplines, geographies, and backgrounds. Beyond the fellowship year, GHC alumni continue to receive training, seed funding, coaching, and networking support.

### Comparison across global health leadership program curriculum design and competencies

In order to facilitate comparisons across the three programs we describe in this paper, we developed a matrix following the curriculum development process in order to compare and contrast each global health leadership program (***Table 1***).

**Table 1:** Comparative Analysis of Global Public Health Leadership Program Curricular Approaches.


CURRICULUM STEP		AFYA BORA	STAR PROJECT	GLOBAL HEALTH CORPS

	*Initial matriculation*	2011	2017	2009

	*Main collaborators*	African Partners: University of Nairobi- Kenya Muhimbili University-Tanzania University of Botswana- Botswana Makerere University-Uganda University of Buea-Cameroon US partners University of Washington Seattle University of Pennsylvania John Hopkins University University of California San Francisco	Public Health Institute (PHI) Johns Hopkins School of Public Health (JHSPH) Consortium of Universities for Global Health (CUGH) University of California San Francisco (UCSF).	150+ partner organizations including Ministries of Health, INGOs, and grassroots organizations across the USA, East and Southern Africa

	*Total enrollment to date*	189 (162 alumni, 27 current fellows)	115 (69 fellows, 46 interns)	1,028 alumni

	*Program duration*	One year fellowship.	Two years for fellows and 3–12 months for interns.	13 months fellowship; career-long support for alumni.

**Steps 1 and 2: Problem Identification and Needs Assessment**	*Problem statement*	Lack of health professionals with leadership and management skills to manage HIV/AIDS programs.	Lack of highly skilled public health workers to fulfill technical roles in local (LMIC focused) global/public health programming.	Lack of diverse leaders in non-clinical public health roles Lack of individuals with the leadership skills, management skills, and professional network necessary for systems change.

	*Current approach (status quo for leadership and management in global health programming)*	**Practitioner perspective** There is an increasing need for health professionals trained to fill in the gaps in leadership and management for the growing number of health-related programs in Africa. Most often these positions are filled by expatriates due to lack of expertise among local health care professionals. **Education perspective** Majority of training programs in Africa focus more on clinical skills as opposed to inculcating leadership skills in their trainees. This has led to well-trained clinicians able to manage patient illnesses but unable to develop and run health programs. Finding leadership and management training opportunities is left up to individuals; many must travel abroad, which creates inequities by limiting training for those with means to travel.	**Practitioner perspective** US residents often fill technical leadership roles for USAID programs. Professionals focus on individual advancement and technical skills; ongoing education is often not planned and relies on apprenticeship. **Education perspective** USAID has invested in internal training modules available to their employees. HIC-based graduate programs are classroom-based and misaligned with priorities of practitioners. Online options are expanding but require time and internet access and are limited in their interactivity, assessment, and evaluation options.	**Practitioner perspective** Non-clinical healthcare workers address critical capacity gaps in public health organizations. Underinvestment in the recruitment, retention, and training of diverse non-clinical health workers results in weak and vulnerable health systems. **Education perspective** Many training and education programs overvalue technical skills and neglect the importance of leadership and management skills-building for achieving global health outcomes. Practitioners are therefore unprepared to respond or adapt to the challenges and systems leadership required to transform complex health systems.

	*Ideal approach (what our programs are aligned to contribute towards)*	**Practitioner perspective** Leadership and management training capacity for health professionals in Africa can be enhanced through collaborations among local governments and implementing partners. **Education perspective** Leadership and management modules tailored to health systems should be integrated to existing health-related curricula. The mode of training should emphasize practical/attachments to enable acquisition of skills and experiences in organizations that also have an immediate impact.	**Practitioner perspective** USAID and other leading international development agencies around the world should provide incentives to invest and share resources with colleagues. Practitioners should access training that will improve their performance and particular work expectations while serving as technical leads and partnering with host country governments. **Education perspective** Relevant educational opportunities should be available broadly to the global health workforce. Teams and organizations should think systematically about supporting ongoing learning. Learning should be targeted towards the needs of individuals and the programs they support.	**Practitioner perspective** Global health organizations should have access to a reliable talent pipeline of diverse, non-clinical staff who have the leadership and management skillsets to successfully lead complex initiatives. **Education perspective** Leaders should be prepared with the leadership and management skillsets to successfully navigate and lead complex change. Leadership and management acumen are considered as essential as technical skillsets.

	*Target learners*	Doctors, nurses, and public health professionals drawn from Ministry of Health, Non-Government Organizations (NGO), Academic Health Institutions.	USAID, Ministries of Health, and NGO managers and technical leads focused on public health programs.	Young professionals (ages 22-30) from diverse national, racial, ethnic, and professional backgrounds.

**Step 3: Goals and objectives**	*Overall goals of the curriculum*	Train African health professionals in evidence-based leadership to implement successful HIV-related and other public health programs. Improve HIV prevention, care and treatment through site-level programs and projects that are part of the fellowship training experience. Support collaborations and networking among current fellows, alumni, and mentors to improve the quality, efficiency, and impact of HIV services locally, nationally, and regionally.	Enable highly skilled public health workers to fulfill technical roles in local global/public health programming Fellows can perform at practicing level, or higher, across all eight core competencies Fellows will develop skills and strategies for knowledge sharing.	Fellows are equipped to be effective leaders who excel in their careers, collaborate with each other, and influence the field of global health.

	*Competency domains*	Leadership attributes Problem solving skills Interprofessional communication: both verbal and oral Accountability and management of health programs, use of data to guide, improve, and advocate for programs, ethical conduct, and research with human subjects Effective writing/proposal writing Translation of research findings to practice, effective project management, principles and tools of human resource management Health policy Budget management	Development practice Communications Cross-cultural practice Capacity strengthening Global burden of disease Equity Ethics Gender	Leadership and management skills are addressed in four areas: systems thinking design thinking authentic leadership collective leadership Technical skills are developed through workplace learning and supplementary programming.

**Step 4: Educational Strategies**	*Educational strategies and pedagogical approaches*	One year fellowship that includes interactive didactic sessions for eight weeks and two 4.5 months attachment site rotations (mentored project oriented rotations) **Fellowship meetings** The fellows attend three fellowship meeting that include orientation, mid-fellowship and final meetings. During the meeting, plenary presentations are made on current issues in Global Health by guest speakers, networking with mentors and alumni and fellows make presentations on their projects. **Didactic sessions** Fellows undergo face-to-face lectures, case studies, and discussions in interprofessional teams. **Attachment site placements** These provide practical skills and offer a chance to implement materials learnt from didactic sessions. During the placement the fellows are supervised by site mentors. Individual fellows are expected to undertake a project that benefits the organisation during the placement under the guidance of mentors. **Alumni engagement** The program offers competitive support for a career development project and attendance of networking forums, including fellowship meetings and conferences.	Two-year fellowship and three-month to one-year internships. **ILP** Development of individualized learning plans (ILP) for each participant at the outset of the fellowship, which is monitored and revised as needed throughout. This plan helps participants organize and anticipate learning needs and develop a holistic and coherent package of learning over the course of the program. **Deliberate Practice** A deliberate practice approach is utilized linking learning with work performance. **Hybrid Mentorship** A hybrid mentorship model was developed and is utilized with peer mentorship groups that focus on core competencies and need-driven sessions on topics informed by the priorities of each particular group as well as general public health challenges, such as the COIVD-19 response. In addition, individual technical mentors are assigned as requested.	**Experiential Learning** Paid 13-month fellowship with placement organizations working on the frontlines of global health in Malawi, Rwanda, Uganda and Zambia. **Co-Fellow Model** We place fellows in pairs—one national and one international fellow—within each organization, to promote cross-cultural learning and collaboration. **Training and Community Building** Fellows meet regularly with their cohort for workshops and community building activities. **Professional Development Fund** Fellows can apply funding to pursue individual learning opportunities. **Mentorship/Coaching** Provided by staff, advisors, alumni, and supervisors. **Career-long Support** Fellows join an alumni community and access ongoing support as they advance in their careers, collaborate with each other, and influence the field of global health.

**Step 5: Implementation**		**Core Curriculum** Consists of eight one-week didactic modules and two workshops (one to two days each) and four distance learning modules Delivery: Based on case studies, Problem based learning approach, Group work and presentations with minimal power-point lectures **Attachment site rotations** Act as areas for experiential learning, sites located in African partner countries including Government facilities (MOH), NGOs, International Health organizations—CDC, USAID. **Post fellowship networking** This is through provision of networking platforms and support of ongoing career activities	**Onboarding** Participant onboarding includes a goals development activity, a baseline competency assessment, and the development of an ILP. Each ILP reflects work-related goals as well as longer-term career goals in a set of specific and individualized learning objectives. The ILP is a contract between the participant, their onsite manager at USAID, and STAR for time and budget allocation for the participant to devote to learning. **Program period** Once onboarding is completed, participants embark on their jobs and complete the activities laid out in their ILP. The STAR learning team also engages them in group mentorship groups and other program-wide opportunities. Each participant has check-ins every six months to monitor progress and to course-correct as needed. **Program wrap-up** Each participant completes an evaluation of the learning experience as well as on each specific activity that they completed. They also complete an endline competency assessment to demonstrate changes in skills and knowledge, particularly across the eight core competencies.	**Recruitment and Placement** We recruit and place a diverse pool of talented young professionals on the front lines of global health. Our leaders fill critical gaps within our competitively selected partner organizations, honing the skills needed to transform health systems throughout their careers. **Leadership Programming** We design and implement a transformative, robust leadership development curriculum. **Community Building** We build a tight-knit community to harness the power of collective leadership. Through summits, trainings, an online portal, and regional chapters, our leaders collaborate across borders and boundaries, amplifying their impact and influence.

**Step 6: Evaluation and Impact**	*Assessment and evaluation approach*	Feedback by fellows on all modules Attachment sites: Completion of bi-weekly journal describing experiences Weekly meeting with site mentors Monthly meetings with primary mentors and peer reviews by fellows Completion of evaluations by mentors and Mentees Final evaluation: Final report Post fellowship: Biannual surveys for alumni—track career progression/long term impact. Feedback from fellows and evaluators used to improve curricula	Evaluation of the program was driven by the development of a theory of change and associated metrics to measure impact. Baseline assessment of participant competence and career goals was conducted for each participant. Regularly monitoring of learning progress was undertaken on an annual basis as well as less formally by project staff. An endline assessment of impact at individual and team levels was also conducted.	Conducted formal impact evaluation in 2018 in partnership with Dr. Amy Lockwood (University of California, San Francisco). These findings informed a new Theory of Impact and a system of impact metrics that measure how GHC impacts our fellows and how our results link to progress in global health. Feedback collected through regular surveys, individual check-ins, and group sessions

	*Impact achieved*	Post fellowship alumni surveys are conducted that cover the following topics: Career development Improvement in performance Professional networking Publications The Afya Bora Consortium has seen positive changes across these indicators and has published more detailed findings elsewhere.	Over the two years since STAR has actively been working with participants, a number of results have been achieved. In addition to onboarding participants on a rolling basis, we have established a learning activities database from which to draw activities for participants at different levels across the core competencies as well as for specific technical and content areas. We have also monitored learning plans and identified gaps in available activities. In response to several gaps, in particular related to public health ethics and gender equity, we have developed tailored modules for STAR participants. More data on the impact of the program is forthcoming as we begin to have participants complete the program and shift further attention towards monitoring and evaluation.	**GHC identifies and supports a diverse community of effective leaders…** 68% of alumni are female, 43% are African nationals 99% of alumni attribute their professional achievements in part to their involvement with GHC **who excel in their careers…** 82% of alumni remain in the fields of global health or human development 35% of our first cohort hold senior-level positions **collaborate with each other…** 61% of alumni have collaborated with another alumnus/a in the past year **and influence the field of global health.** 70% of alumni have spoken publicly, published writing, or participated in advocacy efforts in the past year

	*Scalability/Sustainability*	The main challenge in sustainability has been funding. Training of fellows in their home country/local organizations increases retention and ensures sustainability.	Sustainability of learning budgets is a challenge. Learners still struggle to protect time for learning, even when it is part of their contracts. The learning activities database requires regular updates in order to remain current, which is labor-intensive. Looking ahead, STAR is aiming to identify more ways to align with USAID priorities and engage key partners in sustainable ways.	Partner organizations continue to exhibit high demand for fellows as a proven talent pipeline. GHC’s pathways to scale include leveraging strategic partnerships for: work placements, training, thought leadership, networking and seed funding for alumni initiatives.

	*Lessons learned*	It is possible to implement a leadership fellowship for health professionals that has impact in improving health systems in Africa. Collaboration with government and local partners is key in success The north to south collaborations/networking are important in increasing diversity and opening up opportunities post fellowship	Buy-in and support from partners and funders is critical for equitability and success of such programs. Strong communication links between learning teams and other supervisors can help mitigate challenges.	Value of strategic partnerships Funder strategy—complementary to other global health efforts Alumni investment (network)


#### Steps 1 and 2: Problem Identification and Needs Assessment

The programs described in this paper vary in number of years of implementation, target population, number of participants trained to date, and program model. However, each of the programs were established to address a similar problem: a lack of highly skilled and diverse leaders in LMICs with the leadership and management skills and networks required to address complex global health challenges.

Programs identified that local candidates and candidates from historically marginalized populations were underrepresented in leadership positions due to various barriers, including lack of access to referral networks, underinvestment in recruiting a diverse candidate pool, lack of access to professional training and accreditation, and lack of opportunity for continuous learning and professional development. Furthermore, existing education programs tended to focus on clinical or technical skills and were mostly based in high-income contexts, limiting access to candidates who lacked the time, resources, and ability for travel to these locations. Each program recognized that building a strong and diverse talent pipeline in LMICs would require establishing strategic partnerships and increasing donor investments in locally based leadership training. Additionally, each program recognized the need to expand opportunities to a broad cross-section of leaders, including clinical workers, NGO workers, public sector actors, and young professionals from diverse backgrounds.

The difference in target learners between the programs is the primary driver for the variance in curriculum design: Afya Bora Consortium fellowship focuses on clinical and public health professionals. The STAR project promotes public health managers and technical program leaders. Global Health Corps invests in professionals from interprofessional backgrounds at the early stages of their global health career.

After identifying the target learners, each program reviewed the current approach to recruiting and training leaders in LMICs and then identified opportunities for improvement. The success of this assessment and, ultimately, the implementation of each program depended on close collaboration with strategic partners. At the outset, each program described in this paper engaged a set of key local and global collaborators, including public health organizations, government ministries, donors, and academic partners.

#### Step 3: Goals and Objectives

In step three of the curriculum development process, each program identified objectives for the curriculum, which included expansion of capacity for leadership in LMICs, improvement in work performance and functioning of key public health programs such as HIV, and setting leaders up to become mentors, partners, and contributors to continued knowledge sharing for the field. The main competencies that each program included were essential leadership and management skills, such as communication, strategic partnerships, cross-cultural collaboration, understanding research processes, and evidence generation and use.

The degree of technical capacity development varied between programs. As an example, Global Health Corps emphasizes developing leadership and management skills; technical skills building occurs through on-the-job training, as their cohort of fellows is interdisciplinary and require a broad set of technical tools. Afya Bora Consortium and STAR utilize similar approaches as well, though the balance of leadership versus technical skills differed across programs.

#### Step 4: Educational Strategies

Each program implemented educational strategies to meet the unique needs and requirements of their target learners. Afya Bora Consortium organized around a cohort model, working with a set of participants to complete the core curriculum and to engage in placements or rotations to gain applied experience and skills. STAR utilized an individualized approach to tailor and source appropriate content for each participant’s level and skill needs and also embedded a peer mentorship model to encourage knowledge sharing among participants around core competencies as well as topics of interest to the participants. GHC is a hybrid of these approaches, leveraging a cohort model while also providing coaching and funding so that learners can address individualized skill gaps.

In addition to skills development, all three programs focus on network building. GHC does this not only to support their target learners who are in the early career stage, but also to improve collaboration across global health programs. In addition to utilizing a cohort model, GHC places fellows in pairs—one national and one international fellow—within each organization in order to promote cross-cultural learning and collaboration. STAR emphasizes a peer-to-peer mentorship to harness the extensive experience of many of the fellows and to facilitate collaboration across USAID programs. GHC and Afya Bora Consortium also continue to invest significantly in the alumni of their programs, fostering a community of continuous learning and support.

#### Step 5: Implementation

All three programs included recruitment and an onboarding or orientation process. Afya Bora Consortium and GHC followed a shared schedule of core curriculum. At the end, participants had an exit process or wrap-up phase. Afya Bora and GHC also included ongoing networking and community-building approaches to engage with and support participants after they completed the program. Implementation varied across programs based on the primary educational strategies (e.g., individualized, cohort, hybrid).

#### Step 6: Evaluation and Impact

Finally, each program included an evaluation approach under step six of the curriculum development process. Afya Bora Consortium, STAR, and GHC all developed a theory of change to assess impact. Participant feedback was solicited throughout the participants’ engagement with all three programs, including feedback on specific aspects of the curriculum, as well as an endline reflection on the programs as a whole. Efforts to continue to track longer-term impact and feedback from alumni of these programs also occurs in all three programs. The kinds of impact that the programs aim to achieve include career advancement for participants, improvements in their job performance, networking, and products, such as publications reports and other impacts that they have on the field of global health. Sustainability of all programs is a challenge due to funding. However, changes in pedagogy to support participants learning in-country and applying learning in their own contexts has supported demand, retention, and support for these programs, particularly Afya Bora Consortium and GHC.

Each program has lessons learned, some of which will be explored further in the following section. Some key lessons are that strengthening leadership of a diverse global health workforce is possible. In addition, strong and functional partnerships are essential. Engaging with supervisors and other key stakeholders can help key individuals to whom participants are accountable to understand the value of these programs. Finally, maintaining an alumni network and generally supporting participant engagement with each other is a central factor for success.

## Reflections from practice

We reflect on some of the solutions and innovations that have been developed by the programs to meet the needs of the leadership programs outlined above.

### Contextual factors

Despite different start years and a variety of partners and specific motivations, each of the three programs in this paper aimed to address a similar problem: the lack of appropriately trained local public health professionals to lead and manage health programs in LMICs. The status quo for fulfilling these functions has been a tendency to bring in external experts and to focus on clinical training ahead of public health training for the skilled workers who are trained in LMICs. There has also been a lack of concerted effort to ensure that these leaders contribute to sustainable change—by addressing both challenges related to isolation and burnout, and lack of incentives and support for these workers to build skills and to share knowledge among their teams and within the organizations where they work. In parallel, as the problems faced by health systems around the world continue to become more numerous and complex (e.g., communicable and non-communicable diseases, climate change, rising inequities, and outbreaks, such as Ebola and COVID-19), many have raised the concern that current models for managing these challenges are too fragmented, inefficient, and untenable [18–21]. Against this backdrop, the audacious vision to support capacity strengthening through partnerships, networking, mentorship, and an explicit focus on leadership and management skills needed for the local context has gained traction among donors and host institutions.

To meet these challenges, programs have developed tailor-made curricula for the program and/or for each participant, with a focus on mentorship and coaching, and greater emphasis of the curricula to support transition to practice as opposed to an emphasis on technical knowledge acquisition. These programs focus on supporting individuals who are already dedicated to pursuing leadership roles in global health and supporting them to grow and become continually more effective.

### Innovations

All three programs are different from global health fellowships that focus on HIC settings and learners in that Afya Bora Consortium fellows are trained in their home countries in sub-Saharan Africa, STAR participants work on global programs or are based in the countries where they work, and GHC participants are based in countries where they are working as well. This is aimed at ensuring the trainees identify health systems gaps unique to these settings and assures retention of health professionals in sub-Saharan Africa and other LMICs after training. Despite the programs being offered in sub-Saharan Africa, recruited fellows are drawn from both LMIC and HIC. This innovative approach ensures diversity of fellows, increasing learning and networking opportunities and south-to-south collaborations. The long-term goal for all three programs in their own unique ways is that alumni of these programs will be able to be strong global health leaders working in diverse contexts and roles who can serve as mentors, guides, and future instructors or faculty for subsequent generations of global health leaders. All programs also target an interprofessional set of health professionals. This allows for discussion across different cadres of health professionals and promotes the sharing of both knowledge and perspectives.

### Challenges and limitations for equity, sustainability, and scalability

While overall the innovative curricula proposed have been well received and enjoyed by senior professionals, the implementations of these curricula have presented unique challenges. For example, implementing the individualized learning plans for STAR participants has been a labor-intensive process. It has also not been easy to identify the exact kind of learning activities (right timing, location, cost, etc.) that are appropriate for each participant. Due to project priorities and budget limitations, sustaining and ensuring equity of learning budgets has been a challenge. Given the breadth and depth of cadres of global health professionals that would benefit from learner-centered leadership training, a tailored learning delivery model that meets the needs of the learner is highly recommended. Such a model is best supported by tracking learning needs against competency gaps, which can either be knowledge or skills focused. Many of the learners in these programs continued with full-time work while engaging in an “executive-type” leadership program. Thus, an effective curriculum design needs to incorporate flexibility, a range of learning opportunities, and multi-modes of delivery. However, such an approach is challenged by being resource intensive and by the high likelihood of participant drop-off and difficulties in capturing achievement of learning objectives.

The Afya Bora Consortium fellowship curricula has been designed to accommodate working health professionals, covering a wide variety of required content within a period of one year. Despite the period of the fellowship being short, fellows have been faced with difficulties of getting protected leave from employers to pursue activities for the fellowship. This has ended up limiting some fellows who have wished to complete the curriculum. The other major limitation of the fellowship is funding, and this has affected sustainability.

GHC’s fellowship is composed of participants with varying levels of educational attainment and professional experience, from diverse national, racial, and ethnic backgrounds. Furthermore, fellows work across disciplines in diverse settings during the fellowship year with varying frequency and quality of supervision and support. Designing a cohort-based experiential learning curriculum that responds to the unique needs and abilities of fellows has been a labor and resource-intensive process. GHC has relied on dialogic methods (e.g., case studies, applied learning, small group discussions) over didactic ones in order to engage such a diverse cohort. Staff have also relied on fellow and alumni-designed workshops and resources to supplement the core curriculum and to access new tools and bodies of research. Additionally, it has been important to integrate individualized approaches to meet individual learning needs, such as coaching and mentoring, asynchronous learning (e.g., online learning courses), and access to funds for advanced learning opportunities. Finally, GHC has also found it important to invest in the experiential portion of the fellowship—specifically, the work placements—by sharing resources and investing in the management capacity of fellows’ supervisors. It has, however, been challenging to convene supervisors with regularity. Furthermore, supervisor transitions at partner organizations limit the effectiveness of this intervention.

### Summary of Recommendations

Several approaches are needed in order to strengthen global health curricula and competency frameworks. First, the focus of global health competencies and curricula should be unambiguously linked to local health system needs. This further means ensuring that program leaders and implementors understand the context in which the program and its participants will be operating. Second, emphasizing both individualistic and collectivist approaches to learning is important in engaging and supporting diverse learners. Finally, it is important to emphasize mentorship and opportunities to apply learning in contexts where learners are working in order to provide necessary support to learners and to ensure that learning is integrated into their professional roles.

## Conclusions

There is a need to shift ownership of programs towards local leaders who are currently living and working in settings where the most pressing global health challenges occur. To achieve this goal, curricula need to be tailored to the learner and the context. Strong partnerships and resources—including donor support—are essential to implement and sustain a robust curriculum that addresses core skills for effective leadership and that provides opportunities for experiential and more traditional didactic learning.
